# Three-dimensional evaluation of murine ovarian follicles using a modified CUBIC tissue clearing method

**DOI:** 10.1186/s12958-018-0381-7

**Published:** 2018-08-02

**Authors:** Kyosuke Kagami, Yohei Shinmyo, Masanori Ono, Hiroshi Kawasaki, Hiroshi Fujiwara

**Affiliations:** 1 Department of Obstetrics and Gynecology, Graduate School of Medical Sciences, Kanazawa University, Takara-machi 13-1, Kanazawa, Ishikawa 920-8640 Japan; 2 Department of Medical Neuroscience, Graduate School of Medical Sciences, Kanazawa University, Takara-machi 13-1, Kanazawa, Ishikawa 920-8640 Japan

**Keywords:** Clear unobstructed brain imaging cocktails and computational analysis (CUBIC), Three-dimensional visualization, Tissue clearing, Ovary, Enhanced green fluorescent protein transgenic mice

## Abstract

**Background:**

Recently, we demonstrated the three-dimensional (3D) localization of murine trophoblast giant cells in the pregnant uterus using a modified Clear Unobstructed Brain Imaging Cocktails and Computational analysis (CUBIC) tissue-clearing method and hybrid construct consisting of the cytomegalovirus enhancer fused to the chicken beta-actin promoter (CAG) conjugated enhanced green fluorescent protein (EGFP) transgenic mice. In this study, we applied this method to obtain a transparent whole-image of the ovary and observed the 3D localization of individual oocytes in the developing follicles.

**Methods:**

Ovarian samples were obtained from EGFP transgenic mice and subjected to nuclear staining with propidium iodide (PI) and CUBIC treatment. The detection of double fluorescence signals (green and red) and subsequent reconstruction of 3D images of the whole ovary were performed by light-sheet microscopy and computer programs, respectively.

**Results:**

The ovary became transparent using the CUBIC method and each nucleus of the follicle component cells was uniformly fluoro-stained by PI perfusion. In contrast, EGFP signals were strong in oocytes, whereas those of surrounding granulosa cells were faint. These signal differences in EGFP expression among oocytes, granulosa cells, and theca-interstitial cells produce well-contrasted images of the growing follicles, providing clear information of the 3D localization of individual oocytes.

**Conclusion:**

These results indicate that this procedure is one of the effective approaches to analyze the 3D structure of follicles in the whole ovary.

**Electronic supplementary material:**

The online version of this article (10.1186/s12958-018-0381-7) contains supplementary material, which is available to authorized users.

## Background

The ovary is a specific reproductive organ that contains oocytes. Each oocyte individually matures within its own follicular unit, which contains granulosa cells during folliculogenesis and theca cells from secondary stage onward. The follicle is also an important endocrine unit that produces sex steroid hormones, such as androgen and estrogen. During the development process, follicles become enlarged to prepare for ovulation, accumulating follicular fluid in the cavity concomitant with the proliferation of granulosa and theca cells [1].

It has been established that close interactions among oocytes, granulosa cells, and theca cells are critical for the development of follicles [2]. In order to analyze the relationship between adjacent cell populations, histological evaluation by immunohistochemistry has been one of the useful methods contributing to clarifying the underlying mechanisms. However, it is sometimes difficult to histologically investigate the relationship between oocytes and granulosa cells in well-developed follicles since the oocyte is not centrally located in the follicular cavity, being surrounded by granulosa cells of the cumulus oophorus. These granulosa cells are connected with mural granulosa cells that line the basement membrane throughout the follicular wall [1]. Consequently, the three-dimensional (3D) detection of oocytes in well-developed follicles will provide valuable information in both physiological and pathological conditions.

As a conventional method to obtain 3D images, sequential tissue sections are prepared and subjected to various staining methods, and then 3D images are reconstructed from these images [3–7]. Although these classical techniques are useful to analyze precise structures of various tissues, the preparation of thin and sequential sections of the follicular fluid-containing large follicles is often difficult while maintaining fine structures, and the reconstruction of 3D images, especially around the oocyte, is complex.

To overcome this disadvantage, we used a tissue-clearing technology that can provide 3D images of the entire structure of the follicle without having to make tissue sections. Several groups have developed useful tissue-clearing methods to visualize 3D structures of entire organs, such as Sca*l*e, See Deep Brain (SeeDB), CLARITY, 3D Imaging of Solvent-Cleared Organs (3DISCO), and Clear Unobstructed Brain Imaging Cocktails and Computational analysis (CUBIC) [8–12]. Among them, Sca*l*eA2 was recently applied to the murine fetal ovary and the cleared transparent ovary was subjected to whole-mount immunofluorescence staining, observing the numbers of germ cells in intact ovaries using confocal microscopy and three-dimensional software analyses [13]. We recently reported that CUBIC is an effective method to make the pregnant uterus and placenta transparent. Using hybrid construct consisting of the cytomegalovirus enhancer fused to the chicken beta-actin promoter (CAG) conjugated enhanced green fluorescent protein (EGFP) transgenic mice, we also demonstrated the 3D localization of murine trophoblast giant cells within the maternal uterine muscle layer [14]. Consequently, in this study, we applied this method to the adult ovary to obtain transparent whole images and observed the 3D localization of the individual oocytes in the developing follicles.

## Methods

### Preparation of reagents

CUBIC reagents were prepared as described [12]. CUBIC-1 reagent was prepared as a mixture of 25% weight/weight (*w*/w) urea (Nacalai Tesque, 35,904–45, Japan), 25% weight/volume (*w*/*v*) N, N, N′, N′-tetrakis (2-hydroxypropyl) ethylenediamine (Tokyo Chemical Industry, T0781, Japan), and 15% (w/v) polyethylene glycol mono-pisooctylphenyl ether (Triton X-100) (Nacalai Tesque, 25,987–85, Japan). CUBIC-2 reagent was prepared as a mixture of 50 wt% sucrose (Nacalai Tesque, 30,403–55, Japan), 25% (*w*/*v*) urea, 10% (w/v) 2, 20, 20′-nitrilotriethanol (Wako, 145–05605, Japan), and 0.1% volume/volume (*v*/v) Triton X-100. Both reagents were prepared just prior to use. Before adding Triton X-100, all other chemicals were dissolved with a hot stirrer at 60 °C. Because water evaporation will make it difficult for highly concentrated chemicals to be dissolved, the weight of the solution was monitored frequently, and distilled water was added during a mixing step. After all chemicals except Triton X-100 were dissolved, the solution was cooled to room temperature, and finally Triton X-100 was added.

### Animals

We used nine transgenic female mice expressing EGFP under the control of the CAG promoter (C57BL/6-Tg) [15] and eight wild-type female mice (CD-1/ICR). These mice were sacrificed at the age of 6–12 months. Wild-type mice were purchased from SLC (Hamamatsu, Japan), and all mice were reared under a normal 12-h light/dark schedule. All experimental procedures and housing conditions were approved by the Animal Care and Use Committee of the Kanazawa University Animal Experiment Committee (Approval Number, AP-163714), and all of the animals were cared for and treated humanely in accordance with the Institutional Guidelines for Experiments using animals.

### The CUBIC protocol for the ovary

**Fig. 1:**
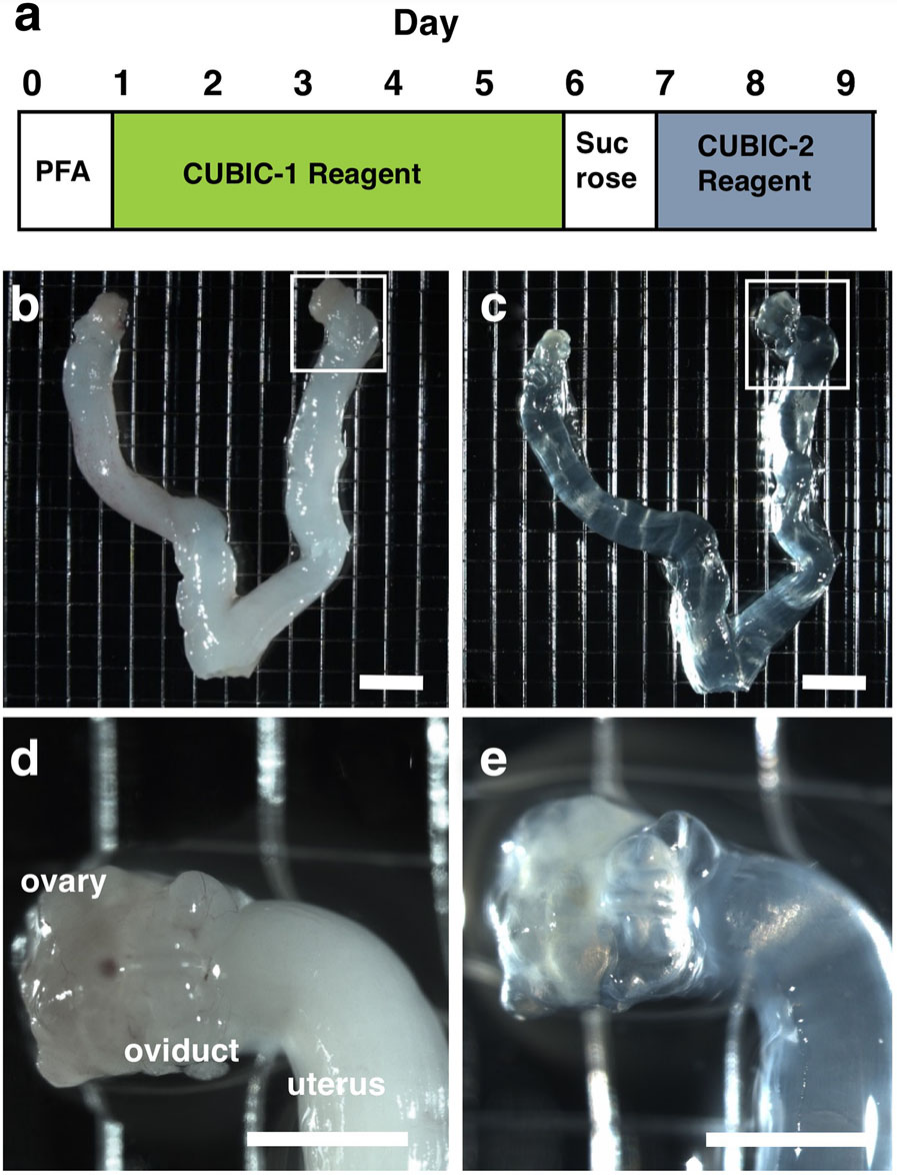
Tissue clearing of the ovary using CUBIC. **a** The procedural protocol of tissue clearing of the ovary using CUBIC. **b**, **c** Bright-field images of reproductive organs of adult wild-type mice before (**b**) and after (**c**) tissue clearing. **d** A higher magnification image within the white square of (**b**). **e** A higher magnification image within the white square of (**c**). Scale bars, 4 mm (**b** and **c**) and 2 mm (**d** and **e**)

CUBIC was performed as described previously with modifications [12, 14] (Fig. 1a). Eight female wild-type mice and six female CAG-EGFP mice were sacrificed by deep anesthesia and transcardial perfusion by 4% paraformaldehyde (PFA) in PBS with or without propidium iodide (PI, Life Technologies), and the bilateral reproductive organs including the uterus, Fallopian tube and ovary were removed. Isolated organs were further immersed in 4% PFA at 4 °C overnight and then incubated in CUBIC-1 reagent at 37 °C for 5 days with gentle shaking. After washing 3 times by PBS with gentle shaking at room temperature for 30 min, the organs were immersed in 20% sucrose in PBS at 4 °C for one day, and incubated in CUBIC-2 regent at room temperature for 2 days.

### Immunostaining

Three adult female CAG-EGFP mice were deeply anesthetized and transcardially perfused with 4% PFA in PBS. To make sections, the ovary was partially dissected, post-fixed by overnight immersion in the same fixative, cryoprotected by overnight immersion in sucrose-containing PBS, and embedded in Optimal Cutting Temperature (OCT) compound (Sakura Finetek, Japan). Sections of 14-μm thickness were made using a cryostat, permeabilized with 0.5% Triton X-100 in PBS, and incubated at 4 °C overnight with rabbit anti-green fluorescent protein (GFP) antibody (Molecular Probe A-11122, 1:500). After being incubated at 37 °C for 2 h with Cy3-conjugated secondary antibody (EMD Millipore AP132C, 1:500) and 1 μg/mL Hoechst 33,342, the sections were washed and mounted.

### Microscopy and image analysis

Bright-field images of the ovaries were taken using a stereomicroscope (MZ16F, Leica). Immunohistochemically stained tissue sections were observed with an epifluorescence microscope (AxioImager A1, Carl Zeiss). Three-dimensional images of transparent organs were acquired using a light-sheet microscope (Lightsheet Z.1, Carl Zeiss) and an epifluorescence confocal microscope (LSM510, Carl Zeiss). Images of the whole ovary were obtained using a 5×/0.16 NA objective lens, and detailed single-cell resolution images were acquired using a 20×/1.0 NA objective lens for the clearing method. Three-dimensional images were analyzed using ZEN software (Carl Zeiss).

## Results

### Tissue clearing of reproductive organs using CUBIC

After tissue-clearing treatment (Fig. 1a), reproductive organs including the ovary became transparent using CUBIC (Fig. 1c and e). Fortunately, the sizes of reproductive organs were not affected by CUBIC (Compare Fig. 1b and d), although it was often reported that the size of organs became larger after tissue clearing [9]. These results suggest that CUBIC is an appropriate method for making the ovary transparent.

### Imaging of the ovary using CUBIC and nuclear staining

**Fig. 2:**
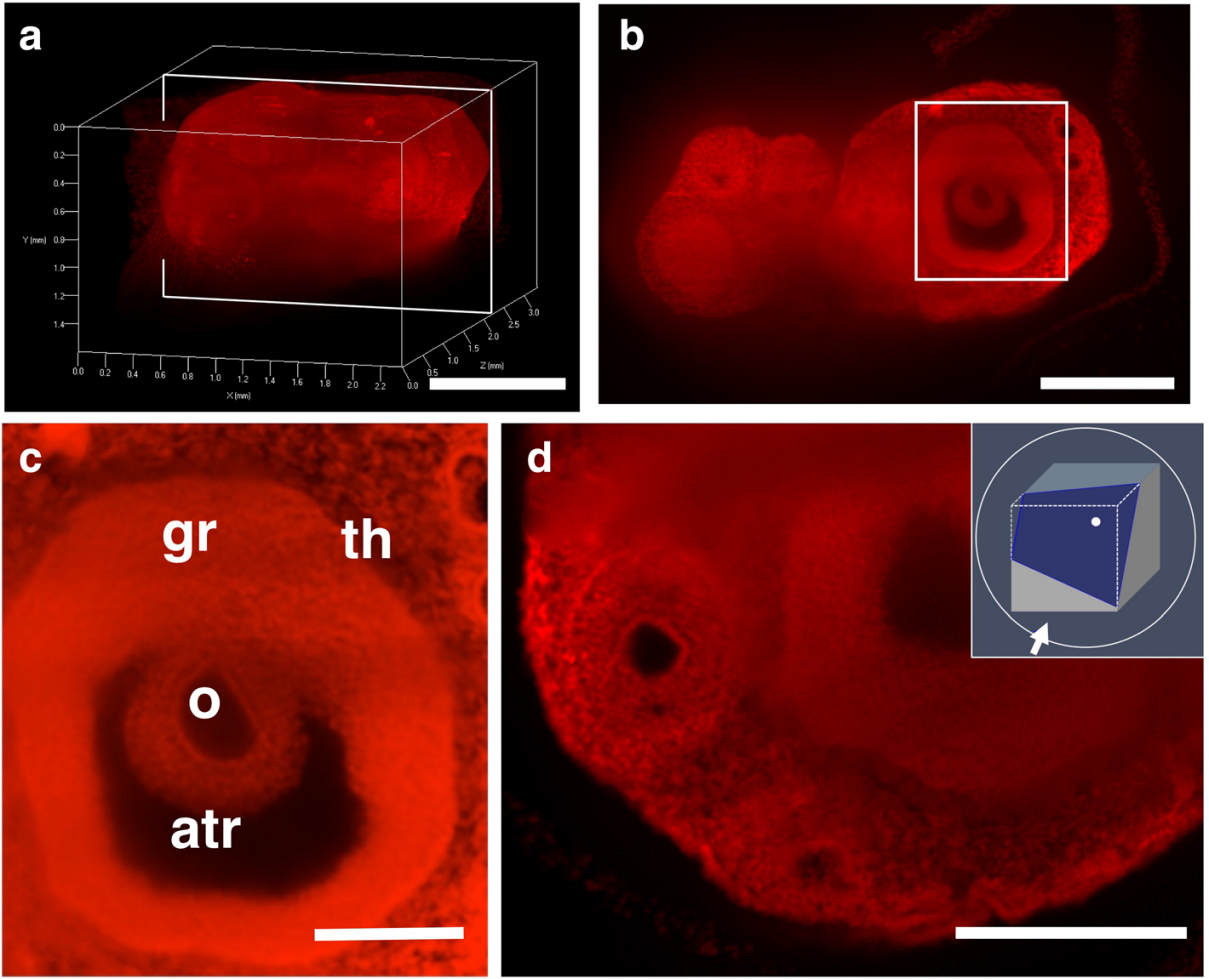
Three-dimensional and cross-sectional images of the ovary stained with PI. **a**-**d** Female mice were transcardially perfused with 4% PFA containing PI, and the isolated ovaries were subjected to CUBIC. Three-dimensional images were taken by light-sheet microscopy. **a** A 3D image of PI signals of the whole ovary. **b** A sectional image within the white square of (**a**). **c** A higher magnification image within the white square of (**b**). **d** A free-angle cross sectional image. gr, granulosa cells; atr, antrum; o, oocyte; th, thecal cells. Scale bars, 1 mm (**a**), 500 μm (**b**, **d**), and 200 μm (**c**)

To visualize fine structures in the ovary, we combined CUBIC with PI nuclear staining and observed PI images by light-sheet microscopy. We successfully detected fluorescence PI signals deep in the ovary and obtained sequential 2D images of the entire ovary with single-cell resolution without having to make tissue sections (Fig. 2b and c). Using the sequential 2D images of PI signals, we were able to reconstruct 3D images of the entire ovary (Fig. 2a). Then, using 3D images of the entire ovary, we were able to reconstruct 2D images of angle-free cross-sections (Fig. 2d). We also clearly observed theca-interstitial structures around antral follicles (Fig. 2c). These results indicate that PI nuclear staining is useful to observe structures in the ovary after CUBIC.

### Imaging of the ovary using CUBIC and EGFP transgenic mice

**Fig. 3:**
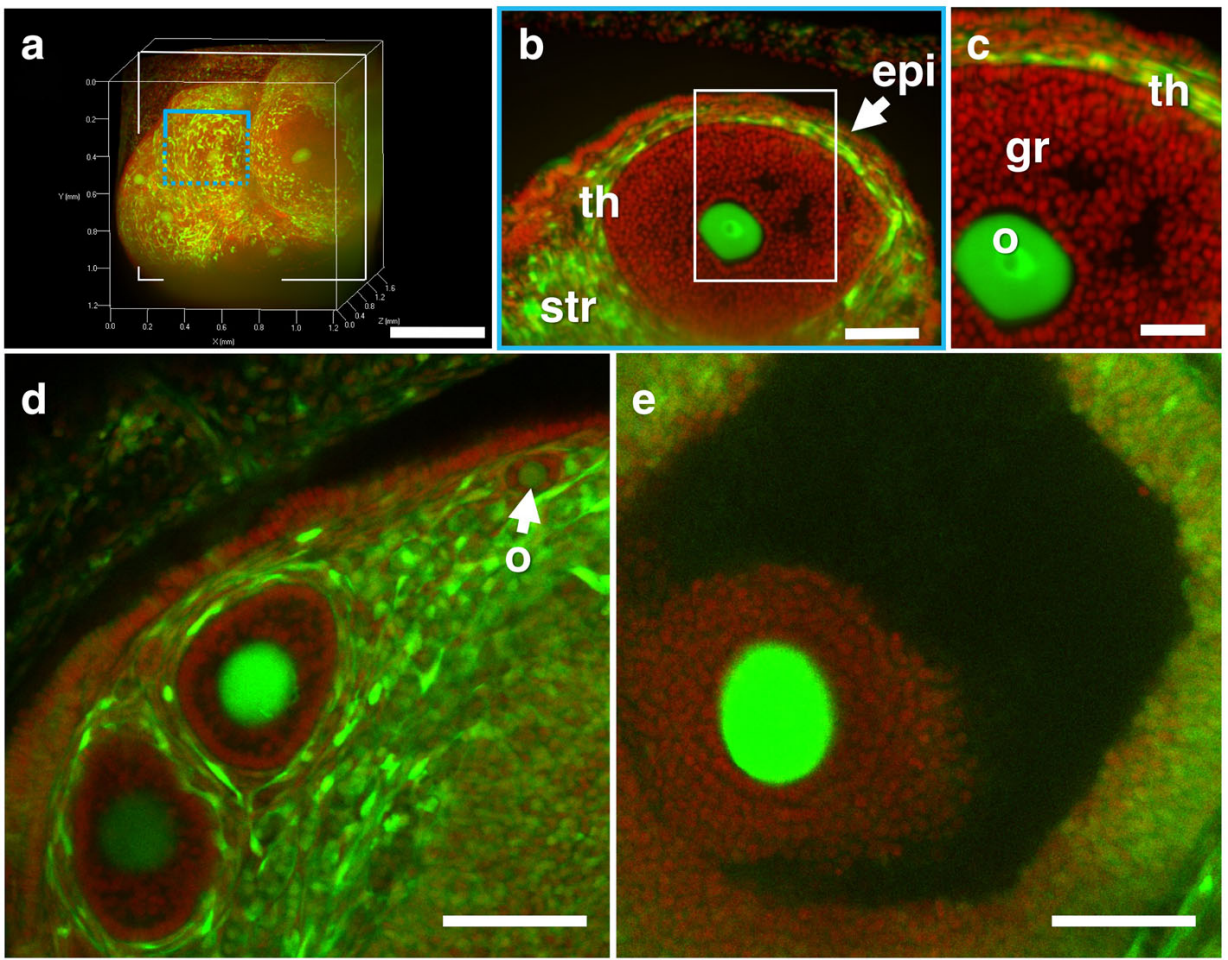
Three-dimensional image of the EGFP-positive mouse ovary. Female EGFP-transgenic mice were transcardially perfused with 4% PFA containing PI. After the isolated ovaries were subjected to CUBIC, 3D and cross-sectional images were taken by light-sheet microscopy (**a**, **b** and **c**) or confocal microscopy (**d** and **e**). **a** A 3D image of EGFP and PI signals of the whole ovary. **b** A sectional image within the blue square of (**a**). **c** A higher-magnification image within the white square of (**b**). **d** Preantral follicles. **e** Mature antral follicle. Note that EGFP fluorescence was strongly visible in the oocyte and ovarian stromal cells, but not in granulosa cells around oocytes. gr, granulosa cells; o, oocyte; th, thecal cells; epi, surface epithelial cells; str, stromal cells. Scale bars, 500 μm (**a**), 100 μm (**b**, **d** and **e**), and 50 μm (**c**)

Although PI staining enabled us to visualize the distribution of the nucleus of each cell, we could barely distinguish the oocytes from other cells. Accordingly, we then used transgenic mice (CAG-EGFP mice) expressing GFP under the control of the CAG promoter, which contains the chicken beta-actin promoter and cytomegalovirus enhancer. The ovary of CAG-EGFP mice with PI treatment was subjected to CUBIC, and 3D images were reconstructed by light-sheet microscopy (Fig. 3a). We clearly observed strong EGFP fluorescence in the oocytes, theca cells, epithelial cells, and stromal cells in 2D images of X-Y cross-sections, whereas the fluorescence signal of EGFP was very weak in granulosa cells surrounding oocyte (Figs. 3b, c, d and e). These differences in EGFP signals among the follicular component cells facilitate contrast imaging that can clearly show the whole shape of oocytes (Fig. 3b and c). In addition, we also obtained high quality images of the preantral and mature antral follicles using confocal microscopy without manual tissue sectioning (Fig. 3d and e, Additional files 1 and 2). Although confocal microscopy unfits the wide-field capturing, these images confirmed that the structure of antral follicle was maintained even though the tissue clearing method was performed (Fig. 3e).

Since a previous study reported that almost all kinds of cells express EGFP in this mouse line [15], we additionally performed immunohistochemistry using anti-GFP antibody to confirm the expression of GFP protein. We detected EGFP protein in granulosa cells even though its expression level was relatively low (Additional file 3).

**Fig. 4:**
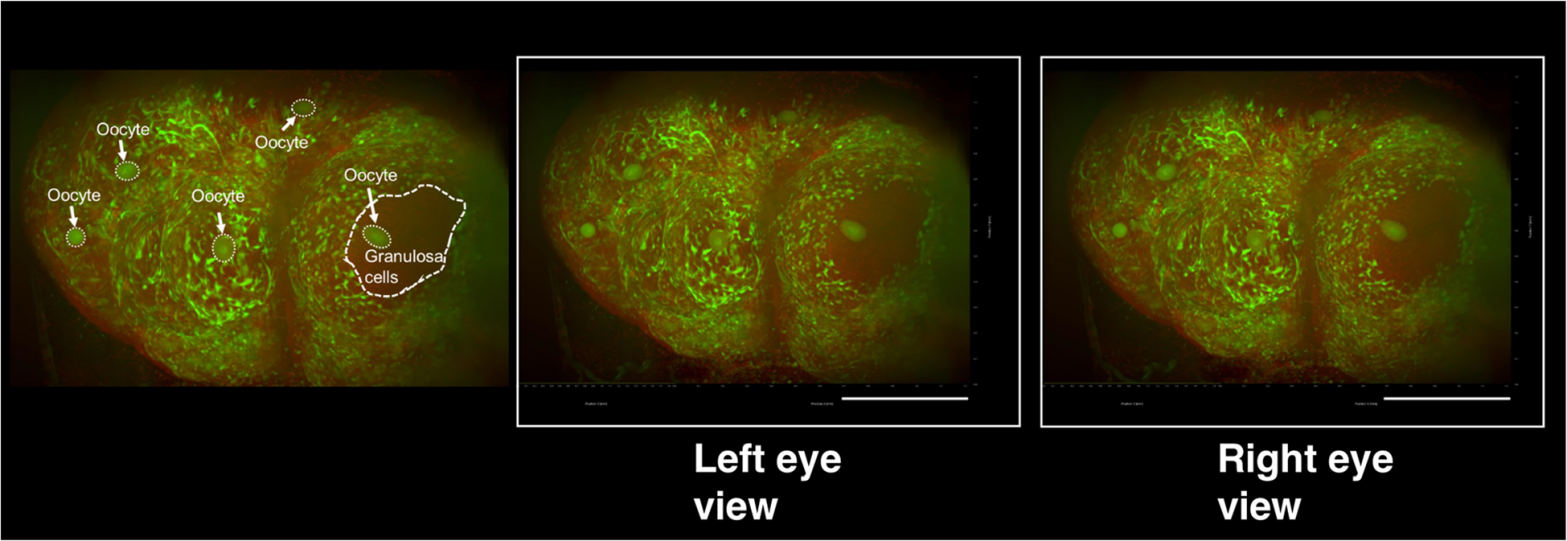
Stereoscopic image of the ovaries of EGFP transgenic mice. Female CAG-EGFP transgenic mice were transcardially perfused with 4% PFA containing PI. After the isolated ovaries were subjected to CUBIC, 3D images were taken by light-sheet microscopy, and then stereoscopic images were reconstructed. Note that the distribution and sizes of EGFP-positive oocytes could be clearly identified. Scale bars, 500 μm


Moreover, using datasets of 3D images, we produced a stereoscopic image, with which 3D locations of EGFP-positive oocytes at various stages could be clearly identified (Fig. 4 and Additional file 4).

**Additional file 4:** A 3D movie of the EGFP-positive mouse ovary. Female EGFP-transgenic mice were transcardially perfused with 4% PFA containing PI. After the isolated ovaries were subjected to CUBIC, 3D images were obtained using a light-sheet microscope. (MOV 2096 kb)


## Discussion

Here, we have shown that 3D images of the whole ovary can be successfully obtained by CUBIC and light-sheet microscopy using EGFP transgenic mice. Previous pioneering studies demonstrated that two key factors of tissue clearing are the homogenization of refractive indices and removal of lipids [16]. Most tissue-clearing methods have been successfully applied to the brain, which is a lipid-rich organ [8–12]. In contrast to the brain, since the ovary does not contain such high lipid levels, it was unclear whether the CUBIC tissue-clearing method was suitable for the mature ovary. Although a recent report demonstrated that Sca*l*eA2 was applicable to the small and immature ovaries before birth [13], in our preliminary experiments using mature ovarian organs, CUBIC provided more transparent than Sca/eA2. Therefore, we applied CUBIC to the mature ovarian organs. Fortunately, the present study clearly indicates that CUBIC is useful to make the ovary transparent.

Conventional histological techniques require a large number of thin tissue sections to create 3D images of the whole ovary. In addition, thin tissue sections of the ovarian tissues are often deformed during experiments because the growing follicles contain follicular fluid. Based on this background, this study indicates that the combination of tissue clearing techniques and light-sheet microscopy can be applied to understand the 3D structure of the whole ovary without having to cut the tissue sections. Especially when CAG-EGFP transgenic mice were used, 3D images of the ovary clearly demonstrated follicles and the corpus luteum at various developmental stages and provided precise information on volumes, numbers, and locations of follicles with single cell resolution. However, since structural information of granulosa cells around oocytes is insufficient in our 3D images, it is relatively difficult to distinguish a primordial or a healthy follicle from a primary or an atretic follicle, respectively, compared with traditional hematoxylin and eosin staining.

Interestingly, although a previous study reported that EGFP is expressed in almost all kinds of cells in CAG-EGFP mice [15], GFP fluorescence in granulosa cells was very weak in CAG-EGFP mice. To clarify the reason for the reduction of EGFP fluorescence in granulosa cells, we immunohistochemically confirmed that immunoreactive EGFP protein was expressed in granulosa cells. Although the precise mechanisms to explain the discrepancy between EGFP fluorescence and EGFP protein expression or differences in EGFP fluorescence intensity among the follicular component cells are still unclear, we consider that there are some differences in the expressing efficiency of the GFP gene under the control of the CAG promoter in reproductive organs. To support this, we observed that there are similar differences in intensity of GFP fluorescence among endometrial epithelial cells, endometrial stromal cells, and myometrial cells within the uterus of CAG-EGFP mice (in preparation). Regardless of the reasons, this characterization of CAG-EGFP mice results in enhancing tissue contrast between various cell types. Since a GFP-positive oocyte is surrounded by granulosa cells bearing faint GFP fluorescence, the size and shape of the oocyte were clearly distinguishable with this method. Since several fluorescent agents such as Cy3 and FITC were reported to be tolerant to CUBIC, we can immunocytologically stain the ovarian cells by fluorescent agents-labeled-antibody during tissue clearing method [17]. Consequently, it is also possible that this method is successfully applied to human ovarian tissues.

Taken together with our previous report using the pregnant uterus and placenta [14], we here propose that CUBIC is appropriate for tissue clearing of reproductive organs. Using 3D images of the whole ovary, 2D images of angle-free cross-sections can easily be reconstructed (Fig. 2d). As described in our previous report [14], we can perform additional immunohistochemical experiments using real tissue sections freshly prepared from the transparent ovary, which correspond to adequate cross-sections that were selected by stereoscopic imaging and the subsequent computed 3D imaging analysis. This advantage offers enormous potential for elucidating the mechanisms of ovarian development and disorders.

## Conclusion

This study successfully demonstrates that the combination of CUBIC, light-sheet microscopy, and EGFP-transgenic mice is useful to observe 3D structures of the whole ovary with single cell resolution. From the 3D images of the whole ovary, we can reconstruct 2D images of angle-free cross-sections without having to make tissue sections. Since the component cells of the ovarian follicles showed different EGFP fluorescence intensities, we also obtained well-contrasted and stereoscopic images and observed 3D localization of oocytes within the individual follicles. Furthermore, a site-specific Cre-loxP recombination system enables us to visualize specific-gene-expressing cells by inserting reporter genes for fluorescent proteins. Consequently, combining our method and a Cre-loxP recombination system, we can theoretically obtain 3D-images of specific-gene-expressing cells in the whole transparent ovary. Based on these advantages, this procedure has the potential to markedly contribute to identifying the mechanisms of ovarian development and disorders in the future.

## Additional files

Additional file 1: Sequential 2D images of X-Y cross-sections of the EGFP-positive preantral follicles. Female EGFP-transgenic mice were transcardially perfused with 4% PFA containing PI. After the isolated ovaries were subjected to CUBIC, sequential 2D images of X-Y cross-sections containing primordial and preantral follicles corresponding to Fig. 3d were taken using a confocal microscope. (MOV 918 kb)

Additional file 2: Sequential 2D images of X-Y cross-sections of the EGFP-positive antral follicle. Female EGFP-transgenic mice were transcardially perfused with 4% PFA containing PI. After the isolated ovaries were subjected to CUBIC, sequential 2D images of X-Y cross-sections containing an antral follicle corresponding to Fig. 3e were taken using a confocal microscope. (MOV 710 kb)

Additional file 3: GFP immunohistochemistry using ovaries of CAG-EGFP mice. Female CAG-EGFP mice were fixed with the transcardial perfusion of 4% PFA. Sections were stained with Hoechst 33,342 and anti-GFP antibody to reveal the expression of EGFP protein. Note that although GFP fluorescence was undetectable in granulosa cells, the expression of immunoreactive EGFP protein was detected. Scale bars, 100 μm. ( 668 kb)

## Abbreviations

3D: Three-dimensional
3DISCO: 3D Imaging of Solvent-Cleared Organs
CAG: Hybrid construct consisting of the cytomegalovirus enhancer fused to the chicken beta-actin promoter
CUBIC: Clear Unobstructed Brain Imaging Cocktails and Computational analysis
EGFP: Enhanced green fluorescent protein
GFP: Green fluorescent protein
O.C.T.: Optimal Cutting Temperature
PFA: Paraformaldehyde
PI: Propidium iodide
SeeDB: See Deep Brain
*v*/v: Volume/volume
*w*/*v*: Weight/volume
*w*/w: Weight/weight
