# Supplementary figures and images for: Three-dimensional evaluation of murine ovarian follicles using a modified CUBIC tissue clearing method
Source: Reprod Biol Endocrinol. 2018 Aug 2;16:72. doi: 10.1186/s12958-018-0381-7 (PMC6091003; doi:10.1186/s12958-018-0381-7)

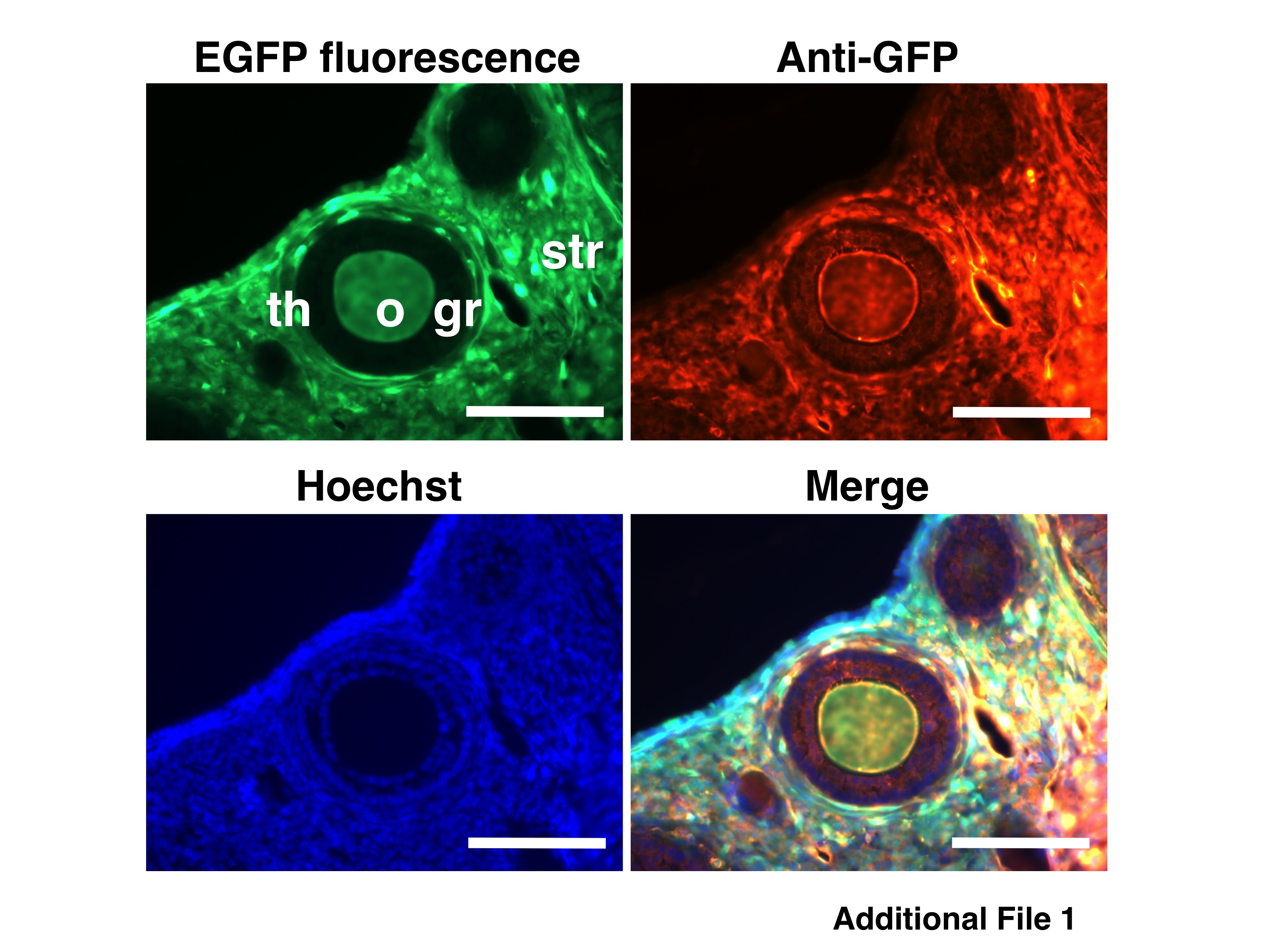

Supplement: Supplementary file 4 — GFP immunohistochemistry using ovaries of CAG-EGFP mice. Female CAG-EGFP mice were fixed with the transcardial perfusion of 4% PFA. Sections were stained with Hoechst 33,342 and anti-GFP antibody to reveal the expression of EGFP protein. Note that although GFP fluorescence was undetectable in granulosa cells, the expression of immunoreactive EGFP protein was detected. Scale bars, 100 μm. ( 668 kb) [file 12958_2018_381_MOESM3_ESM.rmb]
